# Aeroengine Working Condition Recognition Based on MsCNN-BiLSTM

**DOI:** 10.3390/s22187071

**Published:** 2022-09-19

**Authors:** Jinsong Zheng, Jingbo Peng, Weixuan Wang, Shuaiguo Li

**Affiliations:** Aviation Engineering School, Air Force Engineering University, Xi’an 710038, China

**Keywords:** aeroengine, working condition recognition, convolutional neural networks, multi-scale, bidirectional long short-term memory neural networks

## Abstract

Aeroengine working condition recognition is a pivotal step in engine fault diagnosis. Currently, most research on aeroengine condition recognition focuses on the stable condition. To identify the aeroengine working conditions including transition conditions and better achieve the fault diagnosis of engines, a recognition method based on the combination of multi-scale convolutional neural networks (MsCNNs) and bidirectional long short-term memory neural networks (BiLSTM) is proposed. Firstly, the MsCNN is used to extract the multi-scale features from the flight data. Subsequently, the spatial and channel weights are corrected using the weight adaptive correction module. Then, the BiLSTM is used to extract the temporal dependencies in the data. The Focal Loss is used as the loss function to improve the recognition ability of the model for confusable samples. L2 regularization and DropOut strategies are employed to prevent overfitting. Finally, the established model is used to identify the working conditions of an engine sortie, and the recognition results of different models are compared. The overall recognition accuracy of the proposed model reaches over 97%, and the recognition accuracy of transition conditions reaches 94%. The results show that the method based on MsCNN–BiLSTM can effectively identify the aeroengine working conditions including transition conditions accurately.

## 1. Introduction

In the past decades, the time-frequency analysis methods represented by the fast Fourier transform and wavelet analysis [1] and the machine learning methods represented by support vector machines [2] have greatly contributed to the development of fault diagnosis theories and methods. As the heart of an aircraft, it is of great significance for flight safety to achieve efficient monitoring of aeroengines [3]. As technology is constantly being advanced, increasing numbers of parameters can be extracted from the engine to characterize its operating status. Consequently, how this data can be used to monitor engines has been a hot topic of research [4]. Aeroengines often exhibit different fault characteristics under different working conditions, and condition identification is a critical step in engine fault diagnosis [5]. As military aircraft, especially fighter aircraft, often have to make air maneuvers, compared with civilian engines, military engine working conditions change more frequently, and the transition conditions occupy a large proportion of the relevant factors. Therefore, the identification of the transition condition is a very pivotal step in the fault diagnosis of military aircraft engines.

Previously, scholars have carried out research on the recognition of aeroengine working condition. He, D.W. et al. and Zhou, S.M. et al. used a variety of improved support vector machine [6] and support vector description [5] methods, respectively, to study aeroengine working condition recognition. Li, D.Z. et al. used random forest (RF) to study aeroengine working condition recognition [7]. The overall recognition accuracy of these methods is approximately 98%, but the following problems exist: first, most studies have only investigated the identification of the stable working condition of an engine without identifying the transition condition; second, when classifying the conditions, almost all of them only extract the spatial features of the data and disregard the temporal features of the data.

The deep learning method represented by convolutional neural networks (CNNs) provides a new idea for aeroengine working condition recognition. Due to the advantages of weight sharing, robustness, and parameter simplification, CNNs have attained numerous achievements in image recognition, object detection, semantic segmentation, and other fields in recent years. In 2012, Krizhevsky et al. proposed the AlexNet network [8], which consequently sparked interest in deep learning. Since then, more models with more powerful performance such as VGGNet [9], GoogLeNet [10], and ResNet [11] have come out one after another. New models for various specific problems such as R-CNN [12] and YOLO [13] are also emerging and widely used.

How to extract multi-scale features effectively is very important for the classification problem [14]. Hence, Lin, T.Y. et al. proposed the FPN model [15], which comprehensively uses different levels of features containing information of different scales to improve the capacity of the model to identify multi-scale targets. Cai, Z.W. et al. used multi-scale convolution kernels to fully exploit the multi-scale features in the input and improve the feature extraction capability of the model [16]. In addition, the study of attention mechanisms has also greatly uplifted the performance of deep learning models, such as the typical Se-Net [17], CBAM [18], and ECA-Net [19]. Most of these models adaptively adjust the feature weights during error back-propagation to improve the accuracy of the model.

For example, in the field of fault diagnosis, Guo, X. et al. used a hierarchical learning rate adaptive two-dimensional CNN [20] to realize the bearing fault diagnosis research, and the accuracy rate reached 97.9%. Tian, Y. et al. proposed an immune-adaptive deep CNN to realize the fault diagnosis of bearings, with an accuracy rate of 99% [21]. Qian, W. et al. used one-dimensional convolution to conduct fault diagnosis research [22], and the fault discrimination accuracy exceeded 99%. In addition, many scholars have used CNN–LSTM to carry out fault diagnosis research, such as Wei, X.L. et al. and S.-y. Han et al., respectively, using CNN–LSTM to carry out fault diagnosis research on an aeroengine plunger pump [23] and bearing [24], both obtaining higher accuracy.

In this paper, CNN and LSTM are combined to identify the working condition of an aeroengine. First, the excellent feature extraction capability of multi-scale convolution is utilized to extract the spatial features of the data. Subsequently, the BiLSTM is used to extract the time series features of the data to realize aeroengine working condition recognition.

## 2. Basic Theory

### 2.1. Aeroengine Working Condition Recognition

Depending on the power lever angle (PLA), rotating speed, and exhaust temperature of an aircraft engine, its working conditions can be categorized into stable working conditions such as idling, throttling, maximum, and afterburner, and transitional working conditions such as starting, accelerating, decelerating, and switching on the afterburner. Usually, by changing the engine PLA and so on, the engine can be maintained in a certain stable working condition or switched between different working conditions according to certain control laws. Owing to the hysteresis of the control system, for example, when pushing the throttle to accelerate, there is often a certain delay before the engine accelerates to the next stable working condition, while the action of pushing the throttle is often completed in 1~2 s or even less. Therefore, it is inaccurate to simply characterize the PLA as an engine working condition. The aeroengine is working under certain control laws; therefore, the parameters of the aeroengine should be approximated under the same working condition. Based on this, the conditions can be identified using parameters such as the PLA, rotating speed, and exhaust temperature recorded in the flight parameter data, while to identify the transition condition, the change rate of these parameters should be taken into account. The identification of the working condition of an aeroengine can be described as a function of the relationship shown in Equation (1).

(1) $$Cond=f\left(PLA,n,A8,\alpha ,\Delta PLA,\Delta n,\cdots \right)$$

where Cond indicates the engine operating condition, PLA the PLA, n the engine rotating speed including low-pressure rotor rotating speed and high-pressure rotor rotating speed, A8 the tailpipe area, and α the compressor guide vane angle including the variable guide vane curvature or inlet guide vane angle for the fan and high-pressure compressor. ΔPLA indicates the PLA change rate and Δn indicates the rotating speed change rate. Other parameters related to engine operating conditions are included as well.

In fact, owing to the non-linear and complex characteristics of aeroengines, it is very difficult or even impossible to accurately determine the functional relationship shown in Equation (1), but with the help of tools such as machine learning or deep learning, it is feasible to identify the engine working condition by using parameters such as PLA and rotating speed. In particular, with the powerful capability to achieve complex feature extraction of deep learning algorithms, it is possible to identify the working condition of an aeroengine including the transition condition. Considering the characteristics of aeroengine operating parameters, this paper proposes an aeroengine working condition recognition method combining MsCNN and BiLSTM.

### 2.2. CNN–LSTM

The CNN–LSTM model extracts features by comprehensively using the CNN model and the LSTM model to improve the feature extraction capability and robustness of the model. Of these, the CNN model excels in extracting the spatial features of samples, while the LSTM model has a strong ability to extract the temporal dependence of temporal data. The comprehensive use of these two models to extract features allows richer features to be obtained, and the robustness of the model will be stronger [23].

## 3. The Proposed Model

### 3.1. Multi-Scale Convolutional Neural Networks

#### 3.1.1. Multi-Scale Feature Extraction Network

One of the keys to achieving accurate classification is that the extracted features can effectively distinguish different categories. Therefore, it is a very critical step to extract features that can accurately reflect the differences between categories. The core of convolutional neural networks to extract complex features is to use convolution kernels to perform convolution operations. According to [10], a single-scale convolution kernel is only sensitive to features of a specific scale, and it is difficult to extract features of other scales. Therefore, to extract richer features, the model uses multi-scale convolution kernels to extract multi-scale features.

**Figure 1:**
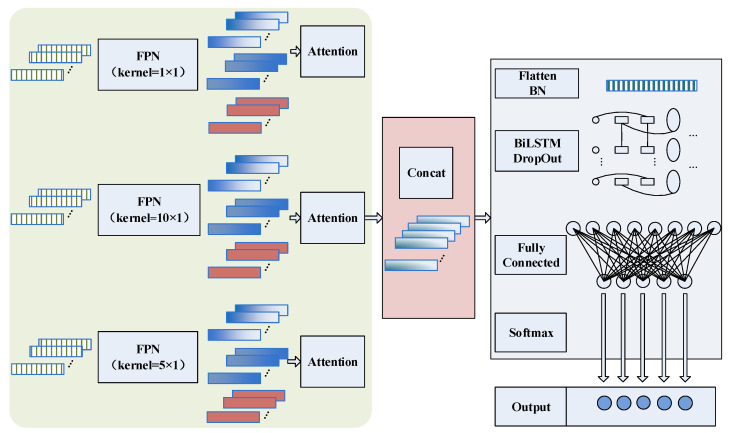
Structure of the proposed model.

**Figure 2:**
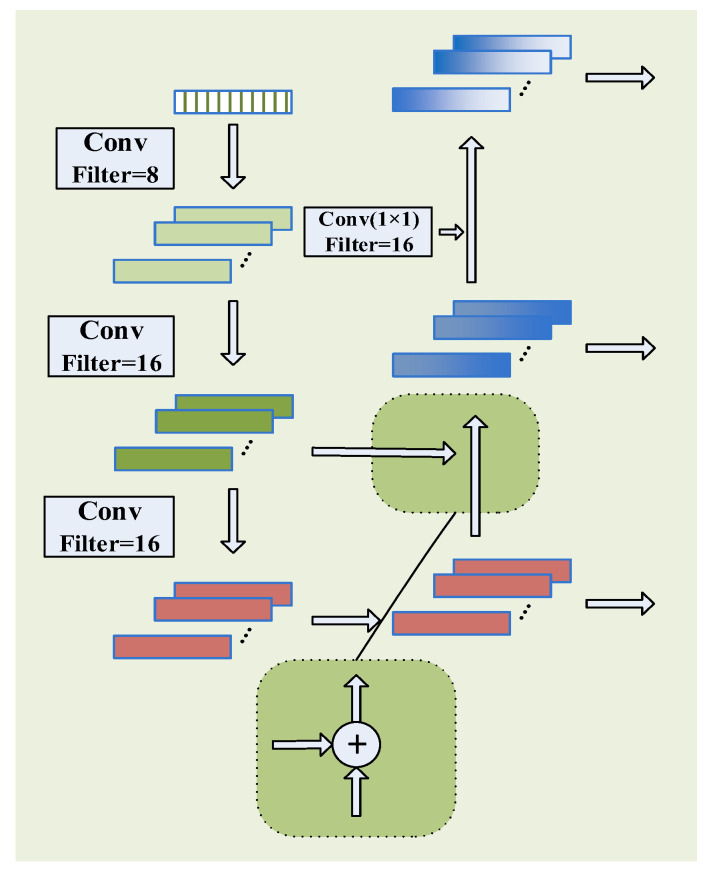
Structure of the FPN feature extraction block.

In addition, the features extracted by the different convolution layers are typically different. Most models use only the features extracted by the deepest convolution kernel as the final features while disregarding the features of other layers, resulting in significant information loss. Therefore, the model incorporates FPNs into multiscale convolutional networks to establish new feature extraction networks for achieving multiscale and multilevel information extraction. The specific implementation method is as follows (Figure 1):

As shown in Figure 2, to achieve multi-scale feature extraction, three convolution kernels with convolution kernel sizes of 1 × 1, 5 × 1, and 10 × 1 are used to perform three layers of convolution, respectively. Considering the small input, the numbers of convolution kernels in each layer are 8, 16, and 16, respectively. In order to realize multi-level information fusion, referring to the FPN network, the features obtained after the third convolutional layer and the features after the second convolutional layer are added element by element, and the features of the second layer are supplemented and enhanced. Then, a convolutional layer with 16 channels and a size of 1 × 1 is used to process the features obtained after the first convolutional layer, so that the number of channels is the same as that of the enhanced second layer feature channels, and then perform element-by-element addition. The enhanced first-layer features are obtained to realize the fusion of multi-level information and make full use of the multi-level features. The features extracted by each convolutional layer are passed through the batch normalization layer and the pooling layer, which improves the calculation speed, reduces the size of the feature, improves the stability and robustness of the model, and uses the activation function to improve the model expression ability.

In addition, to reduce the loss of useful information, the features are not processed using the pooling module after the 1 × 1 convolution kernel. Instead, the extracted features are processed using a 3 × 1 maximum pooling layer in the 5 × 1 and 10 × 1 convolution kernel modules.

#### 3.1.2. Adaptive Weight Correction Unit

In the CBAM, global average pooling and global maximum pooling are used to compress features to calculate channel attention; cross-channel global average pooling and global maximum pooling are used to compress features to calculate spatial attention. In order to compress features within a channel, in Se-Net, intra-channel global average pooling is used. Global average pooling averages the features directly within a channel or at the same location across channels, treating them as equally important. Global maximum pooling also discards a significant amount of other information.

**Figure 3:**
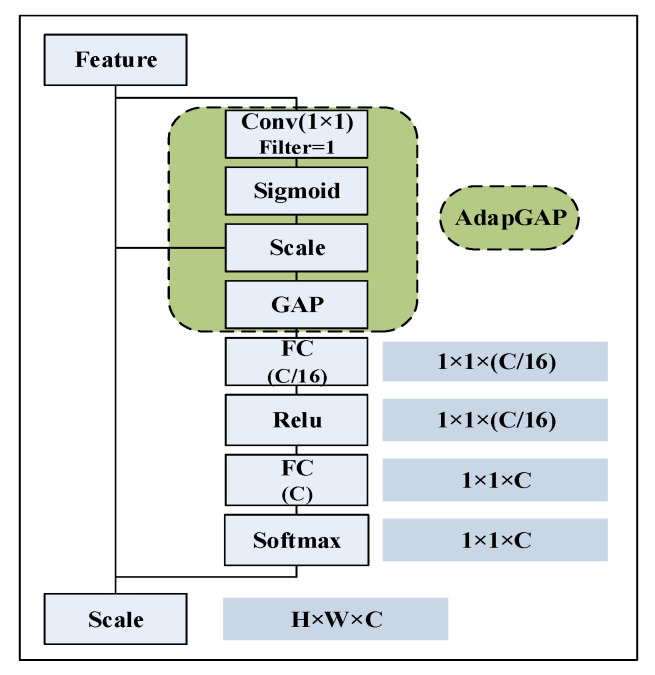
Structure of AdaSE.

To achieve more reasonable feature compression, adaptive squeeze and exciting (AdaSE) and modified spatial attention (MSA) are proposed herein. As shown in Figure 3, when performing in-channel feature compression, AdaSE first uses a 1 × 1 convolution kernel to compress the features into one channel. Then, the weights of this compressed channel at different spatial locations are calculated using the Sigmoid function as the weights at each spatial location of all channels during weighted average pooling, and the weights are assigned to all channels at different spatial locations correspondingly. Finally, the new features with adjusted spatial weights are globally averaged for pooling to achieve global weighted average pooling. The channel attention is learned using the fully connected layer and quantified using the SoftMax function. The spatially corrected features are adjusted using the attention values, and finally, the features with more balanced spatial and channel weights are obtained.

**Figure 4:**
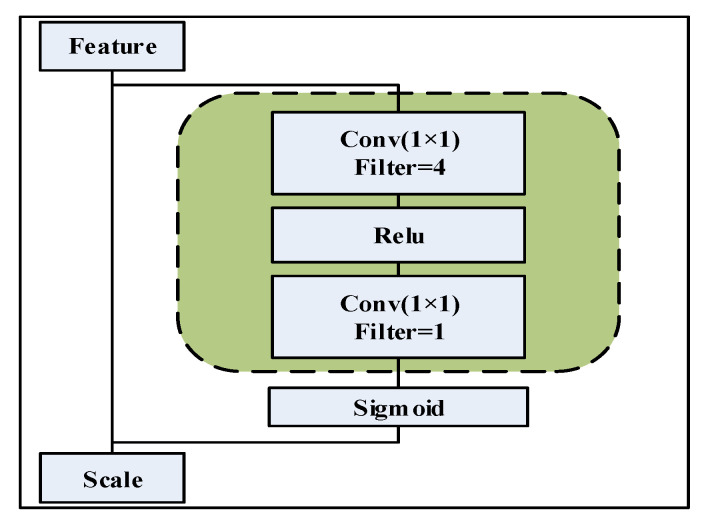
Structure of MSA.

MSA first uses a convolution kernel with four channels and a size of 1 × 1 to compress and fuse the features adaptively and then uses a 1 × 1 convolution kernel to learn the importance of the position in the space. The significance is quantified to obtain the weight distribution of the feature in space. Finally, adaptive correction is performed according to the importance of features, meaning that the model pays more attention to those features that have a great influence on the classification results and suppresses those features that have less influence on the classification results. The specific implementation method is as follows:

As shown in Figure 4, in the spatial weight calculation, the input features are first compressed into four channels by linearly combining them using a 1 × 1 convolution kernel. Then, the features are compressed into one channel using a Relu activation function and a 1 × 1 convolutional kernel that introduces nonlinearity at the same time. The advantages are mainly as follows: (1) in the compression process, the information of all elements is finally used in the extraction of important features by weighted fusion, which retains more useful information; (2) through 1 × 1 convolution, few learnable parameters are introduced, which brings only a small computational overhead; (3) using convolution operations and activation functions, the model can adaptively perform nonlinear adaptive compression of channel features during error back propagation to obtain more reasonable and easily computable features. Finally, the spatially corrected features are put into the AdaSE module to calculate channel attention and perform channel correction.

### 3.2. Overall Structure

As shown in Figure 1, multi-scale and multi-level information is first extracted using the multi-scale feature extraction module, followed by spatial and channel weight correction. The obtained features are Concat fused. Subsequently, the features are flattened using a Flatten layer and imported into the BiLSTM after batch normalization to extract the temporal dependencies of the features. Up to this point, the model has extracted the spatial and temporal dependencies in the original signal. In the end, the classification is achieved using the fully connected layer and the SoftMax function. Focal Loss is used for the loss function to ameliorate the problem of the difficult classification of small samples due to data imbalance, and the final classification results are obtained. The BiLSTM uses 128 units, and the numbers of fully connected layers are 48 and 11, respectively. To enhance the robustness of the model, a DropOut layer with a random drop rate of 0.2 is used after the BiLSTM layer for processing.

The initial learning rate was set to 0.1, with a period of 10 epochs and a decline factor of 0.4. The miniBatchSize was set to 20. The smaller the batch size, the more the model can converge to a flat minimum. However, too small a batch size will result in a very slow model. Therefore, to balance the accuracy and speed of the model, the miniBatchSize was set to 20. To prevent the model from overfitting, the L2 regularization and DropOut strategies were used, with the regularization factor set to 0.0001 and the dropout rate set to 0.2. In addition, when the model did not drop in 50 consecutive validation losses, the iterations were stopped early, and the training was finished.

### 3.3. Main Steps

**Figure 5:**
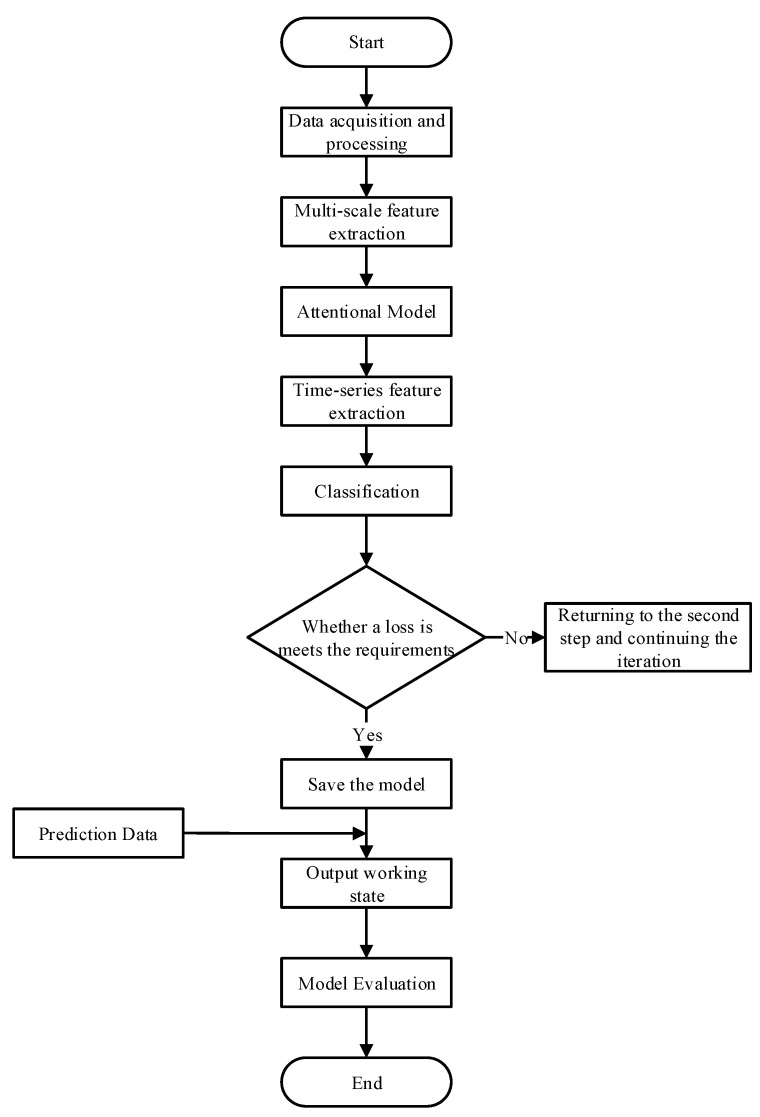
Flow chart of working condition recognition algorithm.

The flow chart of the algorithm when identifying the working condition is shown in Figure 5, which shows the steps of the working condition identification of the aeroengine. Firstly, the desired data are extracted using real outfield flight data, and the obvious outliers are removed from the data. The data are then normalized so that all data are between 0 and 1.

Considering that the variation characteristics of the same parameter are different when the aeroengine is working in the stable condition and the transition condition, when the aeroengine is in the stable condition, its working parameters are relatively steady, and the data fluctuation range is small, while in the transition condition, its parameter changes more prominently, and each parameter changes according to the control law. Therefore, to better identify the working condition of the aeroengine including the transition condition, the engine rotating speed change rate, the PLA change rate, and the tail nozzle cross-sectional area change rate are added to the input signal to enhance the data. The 28 inputs are shown in Table 1.

Then, the MsCNN–BiLSTM model is established according to Figure 1. The model first extracts the multi-scale spatial features of input using MsCNN and adaptively adjusts the features using the attention model. Then, the multi-scale features processed by the attention model are introduced into the BiLSTM to obtain the temporal dependencies. Finally, the fully connected layer and SoftMax classifier are used for classification. During the training process, when the model classification loss meets the requirements or the number of iterations is reached, the training is finished, and the current model is saved. Finally, the test data are imported into the trained model, and the conditions identified by the model are output. The model is evaluated using evaluation metrics such as accuracy and recall, verifying the performance of the model.

## 4. Validation and Analysis

### 4.1. Model Validation

In order to compare the accuracy of different models for the identification of aeroengine working conditions, different models were trained and predicted using the same set of training data and test data, respectively. Accuracy and recall were used as evaluation metrics, where accuracy is Rp and recall is Rr, defined as follows.

(2) $$Rp=\frac{TP}{TP+FP}$$

(3) $$Rr=\frac{TP}{TP+FN}$$

where *TP* represents the number of positive samples predicted as positive samples, *FP* represents the number of negative samples predicted as positive samples, and *FN* represents the number of positive samples predicted as negative samples.

All experiments were repeated 10 times, and accuracy and recall were used as evaluation metrics to compare the typical stable condition and transition condition recognition results. The average of accuracy and recall of the 10 experimental results were taken as the final classification results of the experiments. The experimental results are shown in Table 2 and Table 3, where A indicates acceleration, D indicates deceleration, T indicates throttling, M indicates the maximum state, OFF AF indicates getting the throttle out of the afterburner, and AF indicates the afterburner.

The following conclusions can be drawn from the experimental results:

(1) It is obvious that the proposed model has higher recognition accuracy than BP–ANN, CNN, and BiLSTM models, which have lower recognition accuracy, especially for acceleration and maximum condition recognition accuracy and low recall rate for turning off afterburner recognition. BiLSTM has only a 55.8% recall rate for turning off afterburner recognition.
(2) Compared with the single-scale convolution, the recognition accuracy of the proposed model has been improved by using the multi-scale convolution strategy, especially for the transition condition of acceleration and deceleration, which shows that the multi-scale convolution strategy can effectively extract the features of the engine transition conditions.
(3) The combination of CNN and BiLSTM models resulted in higher model accuracy than when one model was used alone.

**Figure 6:**
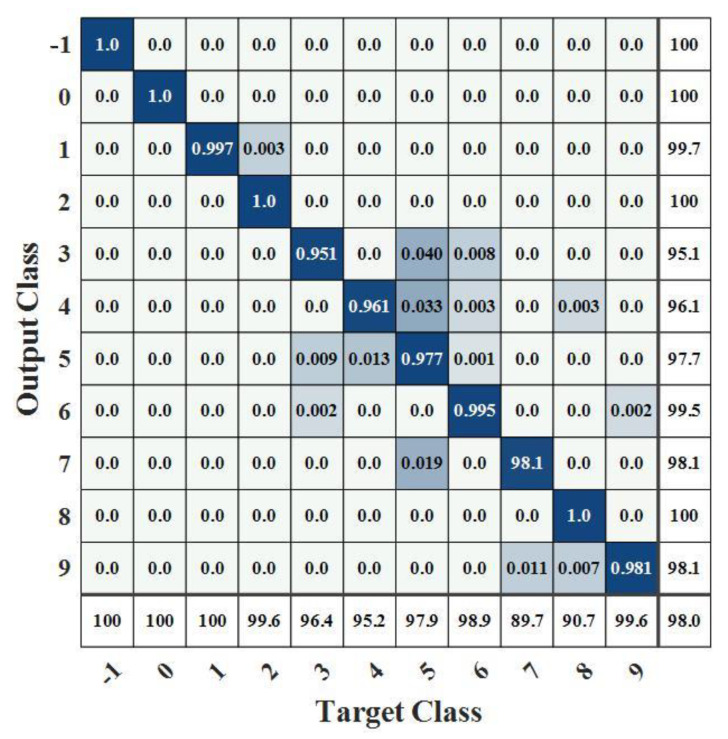
Confusion matrix 1 of working condition recognition.

**Figure 7:**
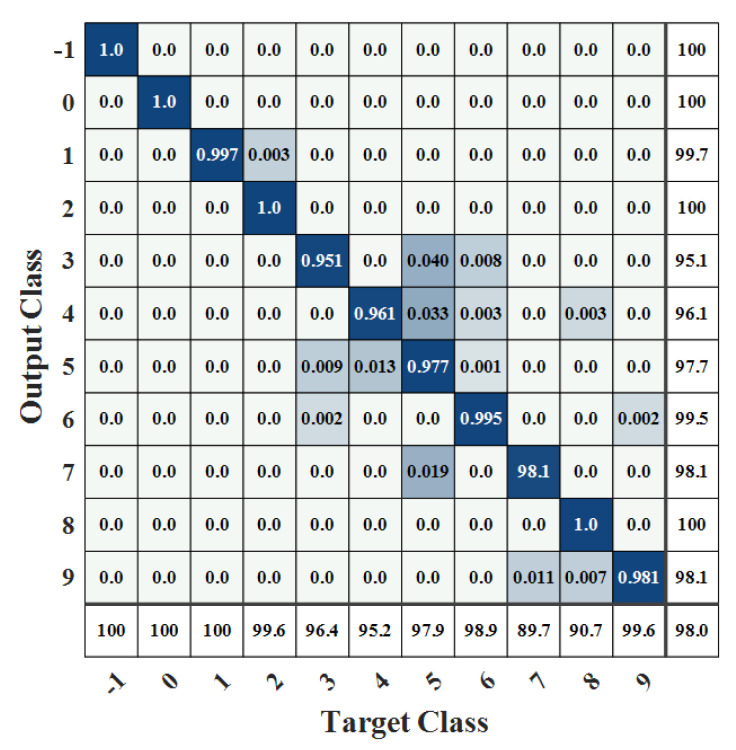
Confusion matrix 2 of working condition recognition.

Figure 6 and Figure 7 show the confusion matrix obtained after model identification. The darker part indicates a greater proportion of the sample. Samples on the diagonal in the matrix indicate the proportion of that sample that is accurately classified. The rightmost side indicates accuracy, and the lower side indicates recall. The correspondence between labels and work conditions is shown in Table 4.

As can be seen from Figure 6 and Figure 7, the recognition accuracy of the model for placing the throttle off and cutting off the engine, unstart, and idling is almost 100%, and the mistakenly recognized samples are mainly concentrated in acceleration and deceleration, mainly because it is difficult to accurately identify when the engine changes from acceleration or deceleration to throttling. In addition, the recognition recall rate for turning on and off the afterburner is low, at only about 90%. The reason is that the parameters of the engine when turning on and turning off the afterburner vary widely, but the changes in rotating speed and temperature are not obvious, making it difficult to distinguish them from the maximum condition and afterburner.

**Figure 8:**
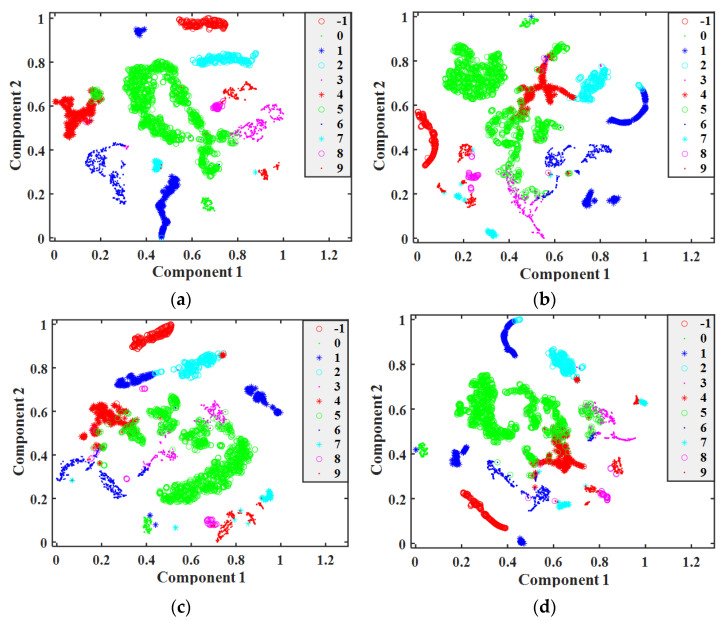
Visualization of the output feature T-SNE reduction: (**a**) the proposed model; (**b**) BP–ANN; (**c**) CNN; (**d**) BiLSTM.

To better analyze the classification effect of each model, the T-Stochastic Neighbor Embedding (T-SNE) method was used to visualize and analyze the final output features of BP-ANN, CNN, BiLSTM, and the proposed models during the testing process. The results are shown in Figure 8.

As shown in Figure 8, it can be seen that the output features of the proposed model are more compactly distributed in each category on the two-dimensional space, with greater spacing between categories and fewer overlapping samples between samples of different categories, compared to BP–ANN, CNN, and BiLSTM. In other words, the proposed model better distinguished the different working conditions and achieved better classification results.

In addition, it can also be seen in Figure 8 that the single-scale CNN and BiLSTM models can also accurately distinguish samples of certain categories, but the overall sample distribution was more dispersed and there were more overlapping samples with less spacing between categories. In particular, the most overlapping samples were found between acceleration (label 3), deceleration (label 4), and throttling (label 5)—a result that also coincides with the results shown in Table 2 and Table 3.

Through the visual analysis of T-SNE, it can be seen that the proposed model can distinguish different categories more effectively than models such as BP–ANN, CNN, and BiLSTM. Therefore, it can be considered that the proposed model can extract more abundant features that can effectively distinguish the differences between categories by fusing the spatial features of different scales and time series features, meaning that it has a higher accuracy. A comprehensive analysis of the model structure and experimental results shows that, compared with traditional methods that can only extract features at a single scale, the proposed model uses a multi-scale convolution strategy to extract richer multi-scale spatial features and extracts features in the time dimension with the help of the BiLSTM model, which fully exploits the hidden working condition features in the flight parameter data, thus achieving the best recognition effect. In addition, due to the inclusion of the attention mechanism, the importance of different features to the classification results is effectively identified, enabling the model to adjust the weights according to the importance of the features, achieving the purpose of improving the model performance.

### 4.2. Analysis of Attention Modules

The core of the attention module is weight adjustment, i.e., improving the accuracy of the model by increasing the weights of features that have a large impact on the classification result and decreasing the weights of irrelevant features. When the SoftMax function is used to quantify the weights, if the attention module cannot learn the importance of the model, it will concentrate a large amount of weight on one or a few features and assign very little weight to the remaining features. Similarly, when the Sigmoid function is used to quantify the weights, if the model does not learn effectively, the weights of each feature tend to be around 0.5.

**Figure 9:**
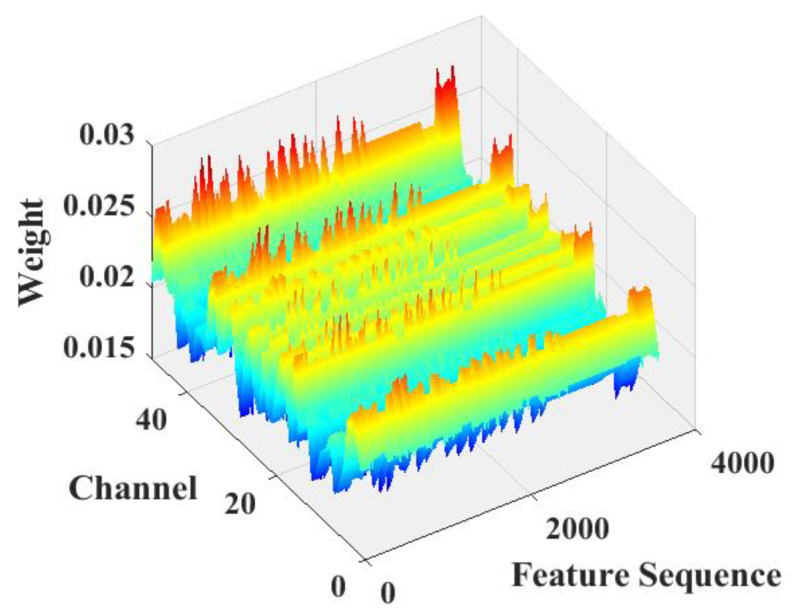
Weight of every channel of AdaSE (Normal).

Figure 9 shows the attention weights output by the SoftMax function of the AdaSE module. Based on the figure, no over-concentration or over-averaging occurred in the weights, indicating that the module effectively learned the importance of each feature and that the spatial importance of the features was effectively differentiated.

**Figure 10:**
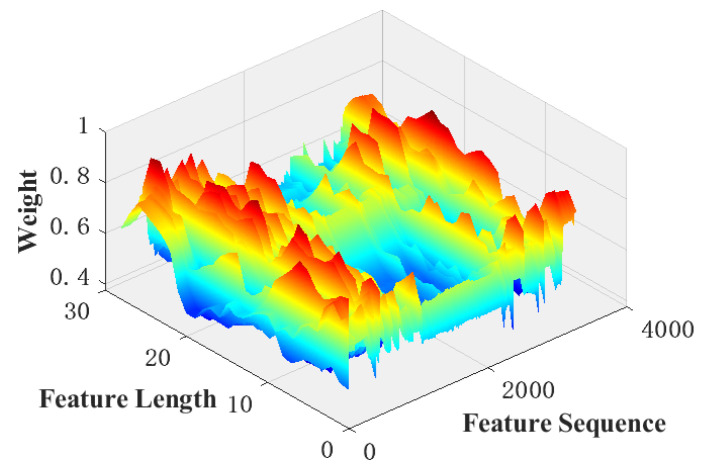
Weight of different spatial position in global weight average pooling.

Figure 10 shows the weights of the different spatial locations when computing the channel attention and performing the global adaptive weighted average pooling. The figure shows that when performing the adaptive weighted average pooling of compressed features, features at different locations within the same channel are not simply summed and re-averaged but are weighted and averaged according to the contribution of the spatial features to the classification result. The features obtained after adaptive weighted averaging pooling contain more information when used to quantify attention weights, thus optimizing the feature compression strategy.

**Figure 11:**
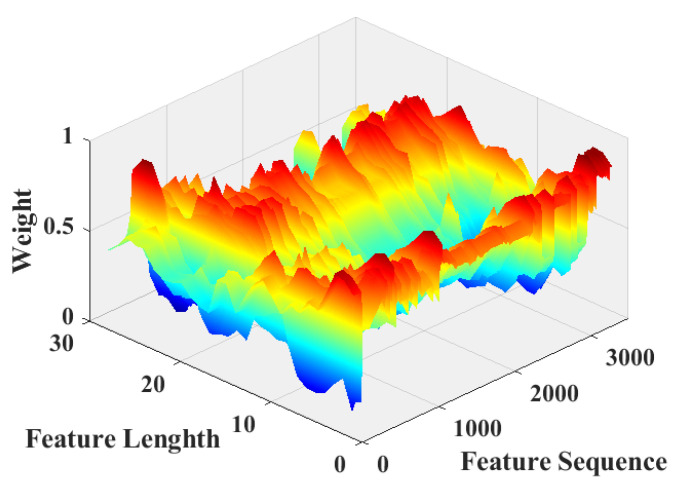
Weight of every spatial position of MSA.

Figure 11 shows the spatial attention weights obtained using the adaptive compression strategy. As shown in the figure, the distribution of weights is not equally distributed around 0.5, which indicates that the module effectively learned the attention weights in space and distinguished the importance of different positions in space.

**Figure 12:**
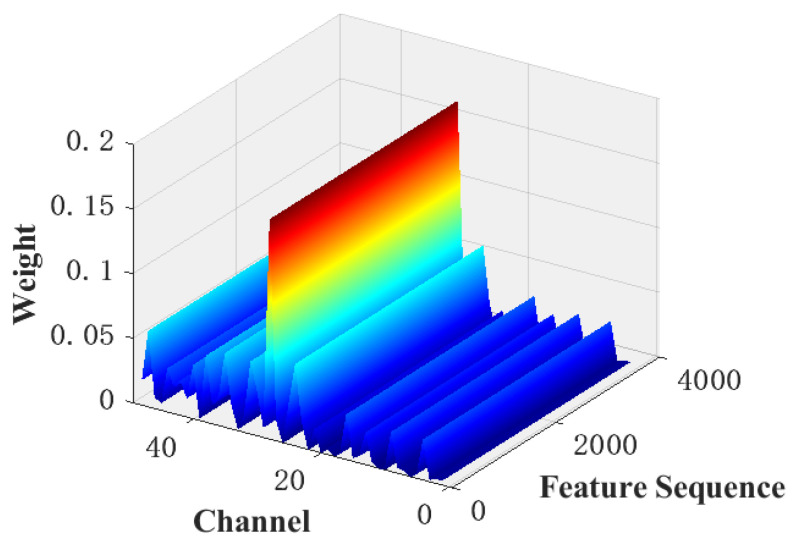
Weight of every channel of AdaSE (Abnormal).

Figure 12 shows the distribution of the attention weights output by the SoftMax function using the proposed attention model when the model is only 83.2% accurate. It can be seen that the model focuses a large amount of attention on one channel, which is much larger than the weights assigned to the other channels, indicating that the model ignores a large number of features on the other channels, thus resulting in an extremely low final classification accuracy.

A comparison of Figure 9, Figure 10, Figure 11 and Figure 12 shows that the submodules of the proposed attention model did not focus significantly on individual features or distribute attention evenly across all features and that they effectively distinguished the importance of features in different channels or spatial locations. The different models were compared with the classification results presented in Table 2 and Table 3. A comparison of the proposed model with the baseline model shows that, after adding the proposed attention module, the recognition accuracy of the model increased from 97.3% to 98%, which fully demonstrates the effectiveness of the proposed attention model. Meanwhile, a comparison between the proposed model and the MsCNN–BiLSTM–SE model shows that the overall recognition accuracy of the proposed attention model was higher owing to the improved feature compression strategy and the combined use of channel and spatial attention.

## 5. Conclusions

In this study, an aeroengine working condition recognition method based on the combination of MsCNN and BiLSTM is proposed. The validity of the model is verified by cases, and the recognition effects of different models are compared. Finally, the attention model is analyzed, and the following conclusions are drawn:

The proposed model can effectively identify the working conditions of an aeroengine containing transition conditions. The overall recognition accuracy is above 97%, and the maximum accuracy of the transition condition recognition reaches 94%.

The recognition accuracy of the proposed model is significantly higher than those of the BP-ANN, CNN, and BiLSTM models, indicating that the proposed model has extracted more effective features to identify the engine working conditions than the other models mentioned in Table 2.

The proposed attention module effectively improves the recognition accuracy of the model with only a minimal increase in the computational overhead, and the accuracy of the proposed model was higher than that of the ordinary Se-Net model, indicating that the proposed adaptive compression and adaptive pooling strategy outperformed the conventional global average pooling compression strategy.

However, the model also has some drawbacks and shortcomings. One is that the accuracy of the identification of turning on and off the afterburner needs to be improved. Second, the established model is complex and time-consuming to train, so a simpler deep learning model for condition identification needs to be further investigated.

## Figures and Tables

**Table 1:** Input signals used in the model.

Variable	Implication	The Employed Data (D(i) = D(j)-D(j-i))
PLA	Power level angle	PLA , PLA(−2) , PLA(−1) , PLA(1) , PLA(2)
n1	Low-pressure rotor rotating speed	n1 , n1(−4) , n1(−3) , n1(−2) , n1(−1) , n1(1) , n1(2)
n2	High-pressure rotor rotating speed	n2 , n2(−4) , n2(−3) , n2(−2) , n2(−1) , n2(1) , n2(2)
α1	Inlet guide vane angle	α1
α2	High-pressure guide vane variable angle	α2
A8	Tailpipe nozzle area	A8 , A8(−2) , A8(−1) , A8(1) , A8(2)
T6	Exhaust gas temperature	T6
s1	Oil supply duty cycle signal	s1

**Table 2:** Recognition Rp of different models in typical aeroengine working condition (%).

Model	Typical Working Condition						Overall Rr
	A	D	T	M	OFF AF	AF	
BP-ANN	92.1	90.2	92.9	97.9	67.4	98.1	94.7
CNN	93.4	97.3	94.5	98.4	83.7	98.5	96.4
MSCNN	94.5	92.9	96.4	98.6	83.7	97	96.7
BiLSTM	86.3	83	84.7	98.3	55.8	98.1	93.8
MSCNN–BiLSTM	94.5	97.3	95.6	99.5	90.7	100	97.3
MSCNN–BiLSTM–SE	95	96.6	96.5	98.7	91.5	99.8	97.6
MSCNN–BiLSTM–MSA	94.5	95.5	96.7	98.9	93	99.6	97.4
The proposed model	96	96.4	97.5	99	91.9	99.6	98

**Table 3:** Recognition Rr of different models in typical aeroengine working condition (%).

Model	Typical Working Condition						Overall Rp
	A	D	T	M	OFF AF	AF	
BP-ANN	86.2	88.9	96.3	95.3	100	92.1	94.7
CNN	89.1	89.6	98.2	98.2	94.7	96.3	96.4
MSCNN	92.8	92.9	96.9	98	87.8	95.9	96.7
BiLSTM	87.1	93	93.5	93.5	100	89.9	93.8
MSCNN–BiLSTM	92.5	91.9	98.3	98.9	100	97.4	97.3
MSCNN–BiLSTM–SE	92.5	93	98.1	98.5	100	97.6	97.6
MSCNN–BiLSTM–MSA	94.3	92.8	97.7	98.4	100	96.7	97.4
The proposed model	95.1	94.7	98.1	99.2	100	97.8	98

**Table 4:** The working condition of aeroengine corresponding to the label.

label	Aeroengine Working Condition
−1	Placing the throttle off and cutting off the engine
0	Unstart
1	Starting
2	Idling
3	Acceleration
4	Deceleration
5	Throttling
6	Maximum
7	Turning on afterburner
8	Getting the throttle out of afterburner
9	Afterburner

## Data Availability

Not applicable.
